# Recombinant characteristics and pathogenicity of a novel PRRSV variant in weaned piglets derived from recombination of three clinical epidemic strains

**DOI:** 10.3389/fvets.2024.1496316

**Published:** 2024-12-13

**Authors:** Yalin Yang, Longshuai Yao, Xinyuan Wang, Xinrong Dong, Gang Wang, Ying Yu

**Affiliations:** ^1^Department of Preventive Veterinary Medicine, College of Veterinary Medicine, Qingdao Agricultural University, Qingdao, Shandong, China; ^2^Shandong Provincial Key Laboratory of Zoonoses, College of Veterinary Medicine, Shandong Agricultural University, Taian, Shandong, China

**Keywords:** PRRSV, NADC30, NADC34, recombination, pathogenicity

## Abstract

**Introduction:**

The recent emergence of PRRSV strains NADC30 and NADC34, along with their recombination with HP-PRRSV-like strains, has added complexity to PRRS control strategies on swine farms. Given the high variability and recombination potential of PRRSV, continuous monitoring of the virus’s clinical epidemiology is essential for effective prevention and control.

**Methods:**

This study isolated a PRRSV variant, designated SDVD-NMG2023, from approximately 65-day-old pigs, showing a mortality rate of around 15% within the herd. The whole-genome, ORF5, and NSP2 sequences of the SDVD-NMG2023 isolate were aligned with 42 reference strains using MEGA software. Recombination analysis was performed using SimPlot software and RDP software. Pathogenicity analysis of SDVD-NMG2023 was conducted in four-week-old SPF Yorkshire piglets.

**Results:**

Phylogenetic and molecular evolutionary analyses revealed a natural recombination event involving the NADC30, NADC34, and JXA1 strains. Piglets infected with SDVD-NMG2023 exhibited mild clinical symptoms, including elevated rectal temperatures in two out of five piglets, as well as cough, mild anorexia, weight stunting, interstitial pneumonia, and thymic atrophy in all cases.

**Discussion:**

The findings indicate that the novel crossbred PRRSV isolate SDVD-NMG2023, derived from three prevalent clinical strains, may induce more unusual clinical presentations compared to those associated with HP-PRRS, albeit still impacting the health of the herd by causing immunosuppression. This study provides critical insights into the emergence of multi-strain PRRSV recombination, particularly between NADC30/34-like and HP-PRRSV-like strains, supporting a more strategic and comprehensive approach to PRRS prevention and control.

## 1 Introduction

Porcine reproductive and respiratory syndrome virus (PRRSV), the causative agent of porcine reproductive and respiratory syndrome (PRRS), has inflicted substantial economic losses on the global swine industry. PRRSV, a small, enveloped, positive-sense single-stranded RNA virus in the genus Porartevirus, family Arteriviridae, order Nidovirales (1), comprises over 11 open reading frames (ORFs)—specifically, ORF1a, ORF1b, ORF2a, ORF2b, ORFs 3–7, ORF5a, and NSP2 (TF). These ORFs encode eight structural proteins and more than a dozen non-structural proteins (NSPs) (2).

Since its initial identification in the United States and Europe in 1987 and 1990, respectively, PRRSV has continued to mutate and recombine within swine herds, leading to its reclassification into two distinct species, PRRSV-1 and PRRSV-2 (1), based on pairwise sequence comparison (PASC) and a 71–77% identity cut-off (3). PRRSV-1 comprises four subtypes, including Pan-European subtype 1 and Eastern European subtypes 2, 3, and 4 (4), while PRRSV-2 is divided into nine lineages (lineages 1 to 9, L1–L9) based on sequence divergence in the ORF5 gene (5).

Infection outcomes of PRRSV-2 vary widely, ranging from asymptomatic to severe, contingent on viral virulence and host factors, including pig age, immune status, and co-infections (6, 7). The initial cases of PRRSV-2 NADC30 and NADC34 were reported in China in 2014 and 2018, respectively (8–10). Both original strains belong to lineage 1 and due to their recombination with highly pathogenic PRRSV (HP-PRRSV)-like strains, numerous NADC30/34-like strains have emerged in swine herds, exhibiting variable clinical manifestations. Infection with either PRRSV NADC30-like strains or NADC34-like strains may induce not only pronounced PRRSV symptoms such as elevated body temperature, elevated fatality rate, and severe pathological alterations (9, 11, 12), but no or mild clinical signs coupled with mild interstitial pneumonia (13–15).

Owing to the substantial variability and recombination tendencies of PRRSV epidemic strains within swine herds, monitoring the virus’s epidemiological trends is critical for effective PRRS prevention and control. To assess PRRSV prevalence in swine farms and investigate its evolutionary features, a novel PRRSV strain (SDVD-NMG2023) was isolated in 2023 from 65-day-old pigs in Inner Mongolia, China. Phylogenetic and molecular analyses indicated that this isolate resulted from natural recombination events among NADC30-like, NADC34-like, and JXA1-like strains. Piglets infected with this isolate exhibited atypical clinical signs, including cough, mild anorexia, weight stunting, interstitial pneumonia, and thymic atrophy. These findings provide valuable insights for the early detection of recombination events among clinical epidemic PRRSV strains, further contributing to the formulation of a national PRRSV control strategy.

## 2 Materials and methods

### 2.1 Sample collection, virus isolation, and identification

In 2023, a cohort of ~65-day-old piglets on an intensive pig farm in Inner Mongolia, China, exhibited respiratory symptoms, including fever, dyspnea, and weight loss. Lung samples from affected piglets were collected and stored at −80°C. Following PRRSV detection, positive samples (1 gram) were combined with Dulbecco’s modified Eagle medium (DMEM, 1 mL) and homogenized using a TissueLyser II (Qiagen, Germany). After centrifugation, the supernatants were filtered through 0.22 μm membranes and seeded into primary porcine alveolar macrophages (PAMs) sourced from specific pathogen-free piglets. After observing 80% of virus-infected cells exhibiting cytopathic effect (CPE), the viruses were harvested, plaques purified using PAM-TANG cells (16), and serially propagated for three consecutive passages, all of which were confirmed to be free of other major swine pathogens. PRRSV N protein detection was performed using indirect immunofluorescence (17), and the isolate was designated PRRSV SDVD-NMG2023.

### 2.2 Viral genome extraction, RT-PCR, and genome sequencing

Viral RNA from the PRRSV strain was extracted from tissue samples using a viral RNA extraction kit (Vazyme Biotech Co., Ltd), following the manufacturer’s instructions. Complementary DNA (cDNA) was synthesized with the Monscript™ RTIII Mix with dsDNase (two-step) (Vazyme Biotech Co., Ltd). Target fragments were amplified by PCR with PrimeSTAR GXL DNA Polymerase (TaKaRa Co., Dalian, China) under the following conditions: pre-denaturation at 94°C for 2 min, 30 cycles (98°C for 10 s, 60°C for 30 s, and 68°C for 2–3 min), followed by a final extension at 72°C for 5 min. The amplified fragments were purified using the CWBIO Gel Extraction Kit (Kangwei Century Biology Co., Ltd.). Using the Takara pMD18-T Vector Cloning Kit, target genes were cloned into the pMD18-T vector per the instructions. Sequencing was performed by Tsingke Biotechnology Co., Ltd. (Beijing, China), and individual fragments were assembled to yield the complete SDVD-NMG2023 genome sequence.

### 2.3 Phylogenetic and lineage analysis of the SDVD-NMG2023

The whole-genome, ORF5, and NSP2 sequences of the SDVD-NMG2023 isolate and 42 reference strains from the GenBank database were aligned with MEGA software (v 7.0). A phylogenetic tree was constructed using the maximum likelihood method in PhyML (v 3.0) with a GTR model of nucleotide substitutions and SPR branch exchange, with bootstrap values calculated from 1,000 replicates. Sequence identity for nucleotides and amino acids between SDVD-NMG2023 and representative PRRSV strains was assessed using Megalign (DNASTAR Lasergene) and MEGA software (v 7.0) (18).

### 2.4 Recombination analysis of SDVD-NMG2023

The recombination patterns of SDVD-NMG2023 were examined using SimPlot software (v3.5.1) and RDP software. Whole-genome similarity mapping in SimPlot identified recombination and breakpoint positions within the parental strains, segmenting the SDVD-NMG2023 sequence into multiple fragments. Parental PRRSV strains with the highest identity and probability were identified through BLAST analysis of the seven defined regions in SDVD-NMG2023 using GenBank.^1^ Additional data on PRRSV, including collection dates and locations, were analyzed to pinpoint potential recombinant parental strains. Concurrently, recombination analysis was conducted using RDP software, employing the RDP, GENECONV, Maxchi, Chimaera, 3 Seq, Bootscan, and SiSscan programs. Recombination events were considered to have occurred when three out of the seven methods indicated the signals. The final assessment of recombination events was based on a combined analysis of SimPlot and RDP results.

### 2.5 Pathogenicity analyses of SDVD-NMG2023 in the piglets

Fifteen four-week-old specific pathogen-free (SPF) Yorkshire piglets, sourced from the vaccine-free swine farm of Sunny-Pig-House in Shandong Province, China, were confirmed to be free of PRRSV infection using an ELISA kit (IDEXX PRRS X3, USA) for PRRSV antibody detection and RT-PCR for PRRSV antigen detection. PCR and RT-PCR testing also verified the absence of Classical Swine Fever Virus (CSFV), African Swine Fever Virus (ASFV), Porcine Circovirus type 2/3 (PCV2/3), Pseudorabies Virus (PRV), *Haemophilus parasuis* (HPS), and *Streptococcus suis* (*S. suis*) antigens. Piglets were divided into three groups (five piglets/group) based on body weight and housed separately in isolated rooms, with access to commercial feed and water *ad libitum* throughout the study. After a one-week acclimation period, two groups were intranasally inoculated with either SDVD-NMG2023 or HuN4 (1 mL per nostril) at a titer of 2 × 10^5^ TCID_50_ in 2 mL DMEM. The control group received DMEM at the same volume via the same route. Clinical symptoms and rectal temperatures were monitored daily and scored using a designated system (19). Blood samples were collected at 0, 3, 5, 7, and 15 days post-inoculation (dpi), and body weight was recorded at 0, 7, and 15 dpi. At 15 dpi, piglets were euthanized with thiopental (20 mg/kg, intravenously) and exsanguinated. Tissues were fixed in 4% paraformaldehyde for histopathological examination, stained with hematoxylin and eosin (HE) to observe pathological changes, and immunohistochemistry (IHC) was performed for virus distribution analysis according to standard procedures (20). This study was approved by the Animal Ethics Committee of Shandong Agricultural University (approval number SDAUA-2023-236) and conducted in accordance with approved animal ethics guidelines.

### 2.6 Detection of viral load in organs and antibodies against N protein

The viremia and viral load of SDVD-NMG2023 in thymus, lung, lymph node, tonsil, and spleen tissues were quantified using an RT-qPCR assay following the protocol described previously (21). Serum samples collected at 0, 3, 5, 7, and 15 dpi were analyzed for PRRSV antibody levels using a detection kit (Beijing JinNuoBaitai Biotechnology Co., Ltd), in accordance with the manufacturer’s instructions.

### 2.7 Statistical analysis

To determine and compare mean values of rectal temperature, body weight, antibody levels, and virus copy number changes among piglet groups, t-tests and multiple comparison methods were applied. All data were presented as mean ± SD. Data analysis was performed using GraphPad Prism 7 software. For multiple comparisons, one-way ANOVA was conducted, followed by Tukey’s or Dunnett’s test as appropriate. The data met the assumptions of the statistical tests used, and statistical significance was set at *p* < 0.05.

## 3 Results

### 3.1 Isolation and identification of the virus

The supernatant from the PRRSV-positive homogenized tissue was filtered and used to inoculate PAMs for virus isolation. Typical PRRSV-induced CPE were observed in infected cells after 78 h. An immunofluorescence assay (IFA) was performed using a monoclonal antibody against the N protein to confirm PRRSV presence, revealing specific fluorescence in infected cells, absent in uninfected controls. These results confirmed the successful isolation of PRRSV, designated as SDVD-NMG2023, with tests confirming it was negative for other major porcine pathogens, including CSFV, PCV2, and PRV.

### 3.2 Phylogenetic analysis and genomic characterization of SDVD-NMG2023

**Figure 1:**
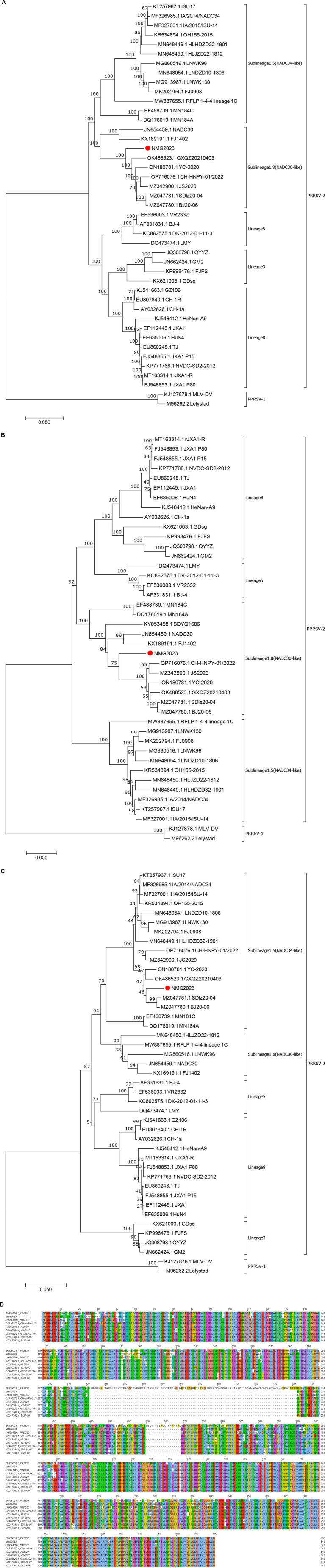
Phylogenetic analysis and sequence alignment of the SDVD-NMG2023 isolate. Phylogenetic trees were constructed based on the whole-genome sequence (A), NSP2 (B), and ORF5 (C) of SDVD-NMG2023 and representative PRRSV strains, and amino acids alignment for NSP2 (D).

The SDVD-NMG2023 genome (GenBank accession no. PQ644630) is 15,021 bp in length, excluding the poly(A) tail. Phylogenetic analysis based on the full genome sequence (Figure 1A), NSP2 (Figure 1B), and ORF5 (Figure 1C) placed SDVD-NMG2023 and representative PRRSVs in distinct lineages. The full genome and NSP2 sequences clustered SDVD-NMG2023 with representative NADC30 strains in lineage L1.8 (Figures 1A,B), while the ORF5 sequence grouped it with NADC34 strains in lineage L1.5 (Figure 1C). Amino acid alignment of NSP2 indicated that SDVD-NMG2023 lacks 131 residues across regions aa 322–432, aa 484, and aa 500–518 (Figure 1D), consistent with NADC30-like strain deletions. Homology analysis revealed that SDVD-NMG2023 exhibited the highest genome sequence identity with NADC30 (89.6%), showing genome identities of 85.1, 86.0, and 86.2% to VR2332, JXA1, and NADC34, respectively. These results reveal that SDVD-NMG2023 exhibits significant genetic diversity compared to previous PRRSV strains, NADC30 and NADC34.

### 3.3 Recombinant analysis of SDVD-NMG2023

**Figure 2:**
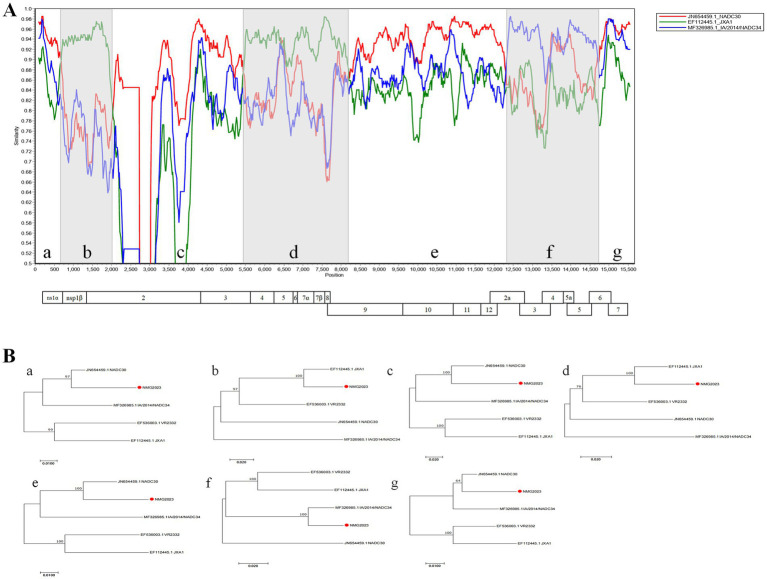
Recombinant analysis of PRRSV SDVD-NMG2023 isolate. (A) Recombination profile of SDVD-NMG2023, analyzed using SimPlot and RDP. The recombination schematic from SimPlot displays the x-axis as PRRSV genome positions and the y-axis as similarity to parental strains. (B) Phylogenetic tree of different regions of SDVD-NMG2023, with SDVD-NMG2023 strains marked by red circles.

To investigate recombination events in SDVD-NMG2023, sequence homology comparisons were conducted between the SDVD-NMG2023 genome and segments of VR-2332, NADC34, JXA1, and NADC30. Nucleotide analysis revealed that SDVD-NMG2023 shares highest homology with JXA1 in the NSP1 and NSP4 to NSP8 regions, with NADC30 in the NSP2, NSP9 to NSP12, ORF6, and ORF7 regions, and with NADC34 in the ORF2, ORF3, ORF4, and ORF5 regions (Figure 2). At the amino acid level, SDVD-NMG2023 displayed highest homology to JXA1 in NSP1 and NSP4, NSP6 to NSP8, to NADC30 in NSP2, NSP3, NSP5 and NSP9 to NSP12, and to NADC34 in ORF2 to ORF6 (Table 1). To validate possible recombination events, RDP 4.10 and SimPlot 3.5.1 software were employed. These analyses confirmed distinct recombination patterns in the SDVD-NMG2023 isolate, supporting the presence of recombination between different parental strains. The analysis identified six recombination breakpoints within the SDVD-NMG2023 genome, located in NSP1, NSP2, NSP3, NSP9, ORF2a, and ORF6 (Figure 2A). These breakpoints segmented the SDVD-NMG2023 genome into seven distinct regions, labeled a, b, c, d, e, f, and g (Figure 2A). MEGA software analysis showed that segments b and d had higher sequence identity with JXA1, while segments a, c, e, and g had higher homology with NADC30 PRRSV, and segment f had higher similarity to IA/2014/NADC34 PRRSV (Figure 2B). To validate these results, RDP 4.0 software was used to re-evaluate the recombination events in SDVD-NMG2023, which confirmed the SimPlot analysis results. Six recombination breakpoints were consistently identified at positions 625 bp, 2019 bp, 5,424 bp, 8,237 bp, 12,312 bp, and 14,704 bp, further substantiating the presence of multiple recombination events within the genome.

**Table 1:** Sequence comparison results between PRRSV SDVD-NMG2023 and other reference strains.

PRRSV strains (GenBank accession No)	VR2332 (EF536003.1)		NADC34 (MF326985.1)		JXA1 (EF112445.1)		NADC30 (JN654459.1)	
Nucleotides or Amino acids	nt%	aa%	nt%	aa%	nt%	aa%	nt%	aa%
Complete genome	85.1	–	86.2	–	86.0	–	89.6	–
5’UTR	94.7	–	94.1	–	89.9	–	97.4	–
ORF1a	83.1	82.2	80.9	80.9	85.8	85.1	86.5	87.7
ORF1b	86.8	95.0	88.1	95.6	86.7	95.7	94.1	97.7
Nsp1	86.0	87.7	84.5	84.6	90.7	94.0	86.1	85.1
Nsp2	78.3	73.8	77.2	72.4	78.4	74.4	87.9	85.4
Nsp3	85.1	92.6	85.0	92.6	86.7	93.1	86.7	94.8
Nsp4	88.6	92.6	84.0	92.6	94.8	97.5	83.3	91.7
Nsp5	87.8	90.0	84.9	89.4	92.2	91.8	89.8	92.4
Nsp6	93.8	93.8	85.4	87.5	97.9	100	91.7	93.8
Nsp7	86.6	86.9	80.8	84.9	94.5	94.2	81.0	82.2
Nsp8	90.4	93.3	88.1	86.7	93.3	93.3	85.2	88.9
Nsp9	87.1	95.6	87.7	95.6	86.6	96.4	92.6	96.7
Nsp10	84.9	95.2	89.9	98.2	84.7	94.3	94.7	98.6
Nsp11	88.9	94.2	87.6	94.6	89.1	96.4	96.1	99.1
Nsp12	87.9	92.9	85.5	89.6	89.0	95.5	95.2	96.8
ORF2	87.8	85.6	95.5	93.4	86.4	84.0	85.7	84.8
ORF3	83.5	82.4	93.5	92.9	83.7	81.6	85.9	83.9
ORF4	87.3	87.7	94.4	96.1	85.7	87.7	94.0	95.0
ORF5	86.1	86.1	95.2	94.0	85.6	87.6	87.1	90.5
ORF6	89.0	93.7	93.7	95.4	87.6	93.1	94.5	95.4
ORF7	90.9	91.1	93.8	91.1	88.7	88.7	96.0	93.5
3’UTR	87.0	–	94.0	–	89.5	–	98.0	–

### 3.4 Clinical features of the piglets infected with SDVD-NMG2023

**Figure 3:**
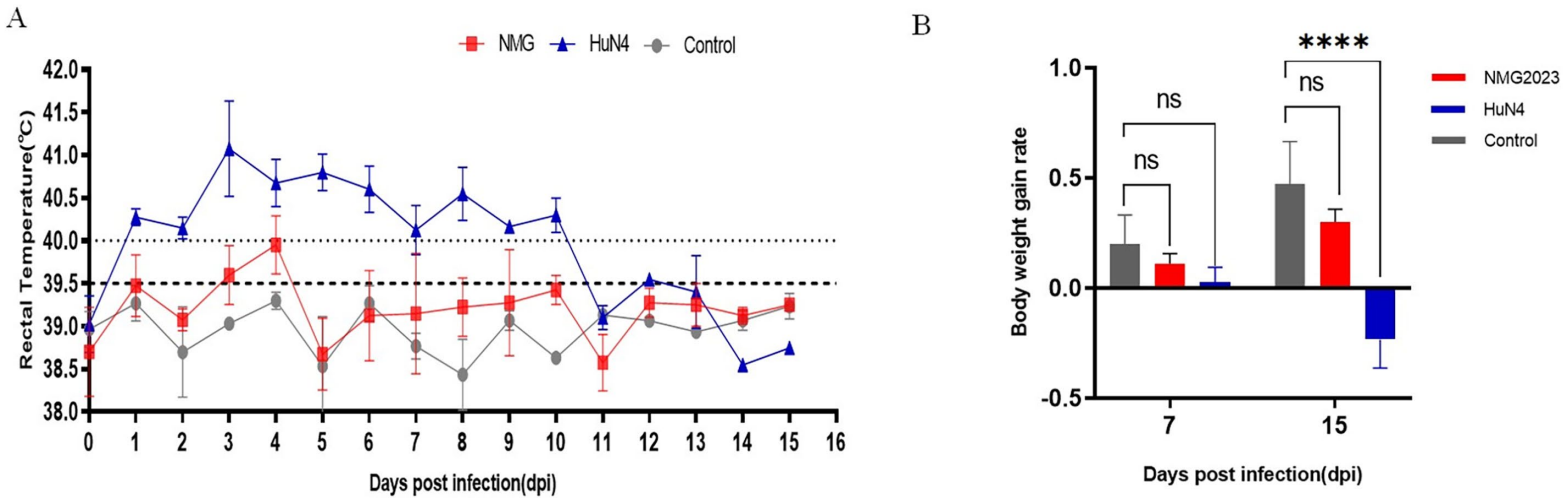
Rectal temperature, weight gain, and thymus atrophy in piglets. **(A)** Rectal temperature profiles of piglets infected with SDVD-NMG2023, HuN4, and uninfected controls. The fever threshold is set at 40.0°C. **(B)** Daily weight gain rate comparison among SDVD-NMG2023-infected, HuN4-infected, and uninfected piglets. Significant differences between HuN4-infected and uninfected groups are indicated at ****p < 0.0001. Data are presented as mean ± standard deviation (error bars).

The HP-PRRSV HuN4 strain, with well-documented virulence, was used as a benchmark to evaluate the virulence of SDVD-NMG2023. Following SDVD-NMG2023 infection, piglets exhibited milder clinical signs compared to those induced by HP-PRRSV. The average rectal temperature peaked at 40.1°C at 4 dpi and subsequently remained below 40°C until the end of the experiment at 15 dpi (Figure 3A). Mild anorexia and cough were observed in the infected piglets from 5 dpi to 15 dpi, along with signs of weight faltering at 7 and 15 dpi compared to controls (Figure 3B). In contrast, piglets infected with HuN4 displayed typical HP-PRRS clinical signs, including fever, cough, mild anorexia, and weight loss. Rectal temperature increased from 1 dpi to 11 dpi, reaching a peak of 41.9°C at 3 dpi. In the negative control group, body temperature remained within normal limits (Figure 3A).

### 3.5 Macroscopic and histopathological lesions of the thymus and lung

**Figure 4:**
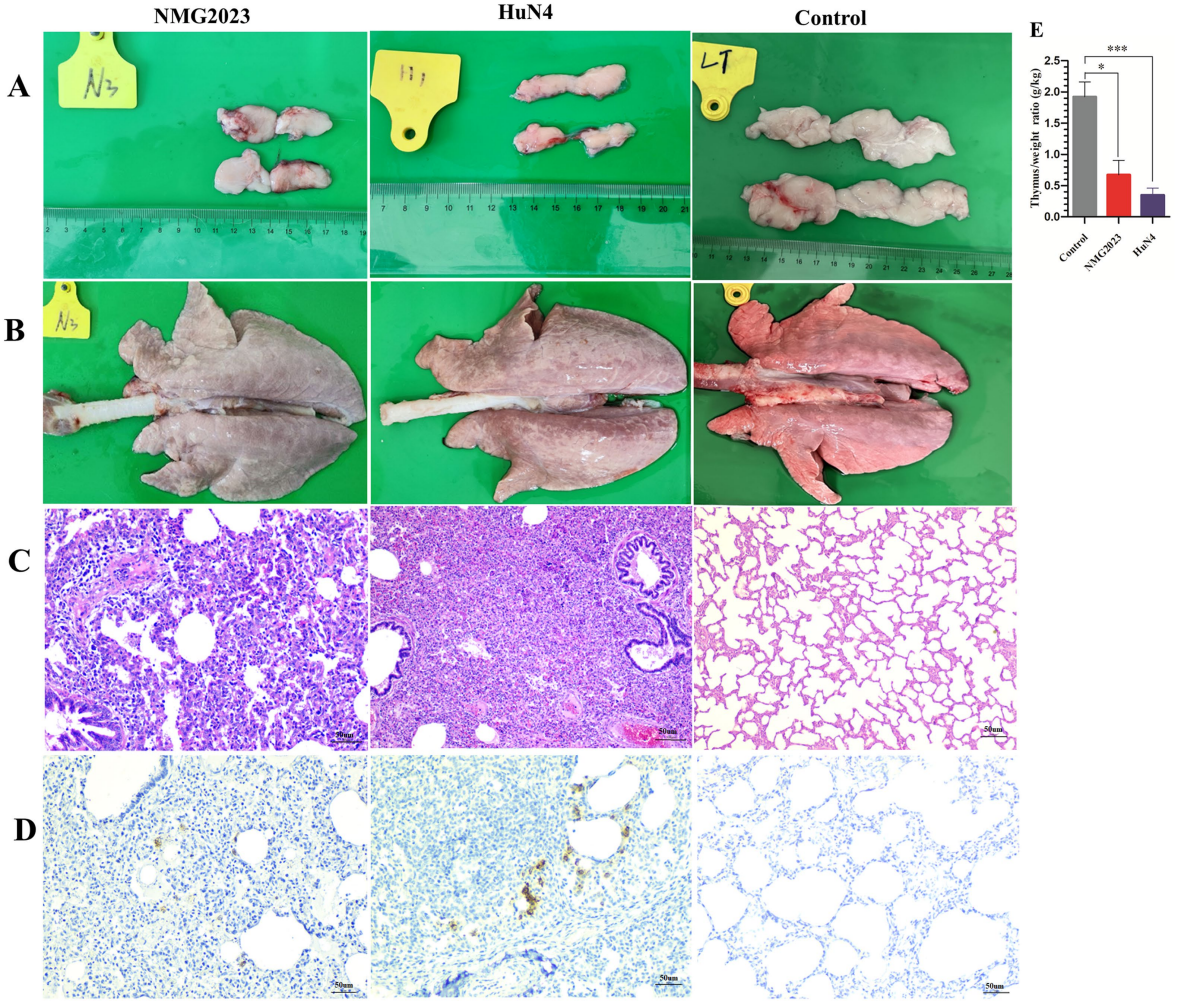
Pathological lesions in the thymus and lung. All piglets were euthanized at 15 dpi. **(A)** Thymus atrophy was observed in both SDVD-NMG2023- and HuN4-infected piglets, while the thymus in uninfected piglets appeared normal. **(B)** Lung consolidation was present in SDVD-NMG2023-infected piglets, whereas lungs in uninfected piglets showed no abnormalities. **(C)** Histopathological examination of lungs in SDVD-NMG2023-infected piglets revealed substantial lymphocytic infiltration, contrasting with the normal lung tissue observed in uninfected piglets. (D) PRRSV-positive signals in the lungs of piglets infected with SDVD-NMG2023 and HuN4 were detected, with uninfected piglet lungs remaining unaffected. (E) Thymus-to-body weight (g/kg) ratios in SDVD-NMG2023-, HuN4-infected, and uninfected piglets. Significant differences between SDVD-NMG2023-infected and uninfected groups are indicated at *p < 0.05, and between HuN4-infected and uninfected groups are indicated at ***p < 0.001. Data are presented as mean ± standard deviation (error bars).

An autopsy performed at 15 dpi revealed marked reductions in the thymus size and weight in both SDVD-NMG2023- and HuN4-infected piglets compared to controls. The thymus weight ratio was significantly lower in the infected groups (SDVD-NMG2023, *p* < 0.05; HuN4, *p* < 0.01) than in the control group, which exhibited no thymic atrophy (Figures 4A,E). Piglets infected with SDVD-NMG2023 displayed mild to severe interstitial pneumonia with inflammatory cell infiltration in the alveolar walls (Figures 4B,C, SDVD-NMG2023 group). In contrast, HuN4-infected piglets exhibited severe lung pathology, including edema, hemorrhage, and lung consolidation (Figure 4B, HuN4 group), with extensive inflammatory cell infiltration in both alveoli and alveolar walls in two out of five piglets (Figure 4C, HuN4 group). Only minimal inflammatory cells were observed in the lungs of uninfected control piglets, which showed normal lung structure (Figure 4C).

PRRSV antigen distribution in the lungs was assessed using immunohistochemistry (IHC) with a monoclonal antibody against PRRSV N protein. Both SDVD-NMG2023- and HuN4-infected piglets displayed PRRSV-positive signals localized in or adjacent to the alveolar walls, primarily in cells with macrophage-like morphology. No positive signals were detected in the control group (Figure 4D).

### 3.6 Viral load in serum and organs and antibody response detection

**Figure 5:**
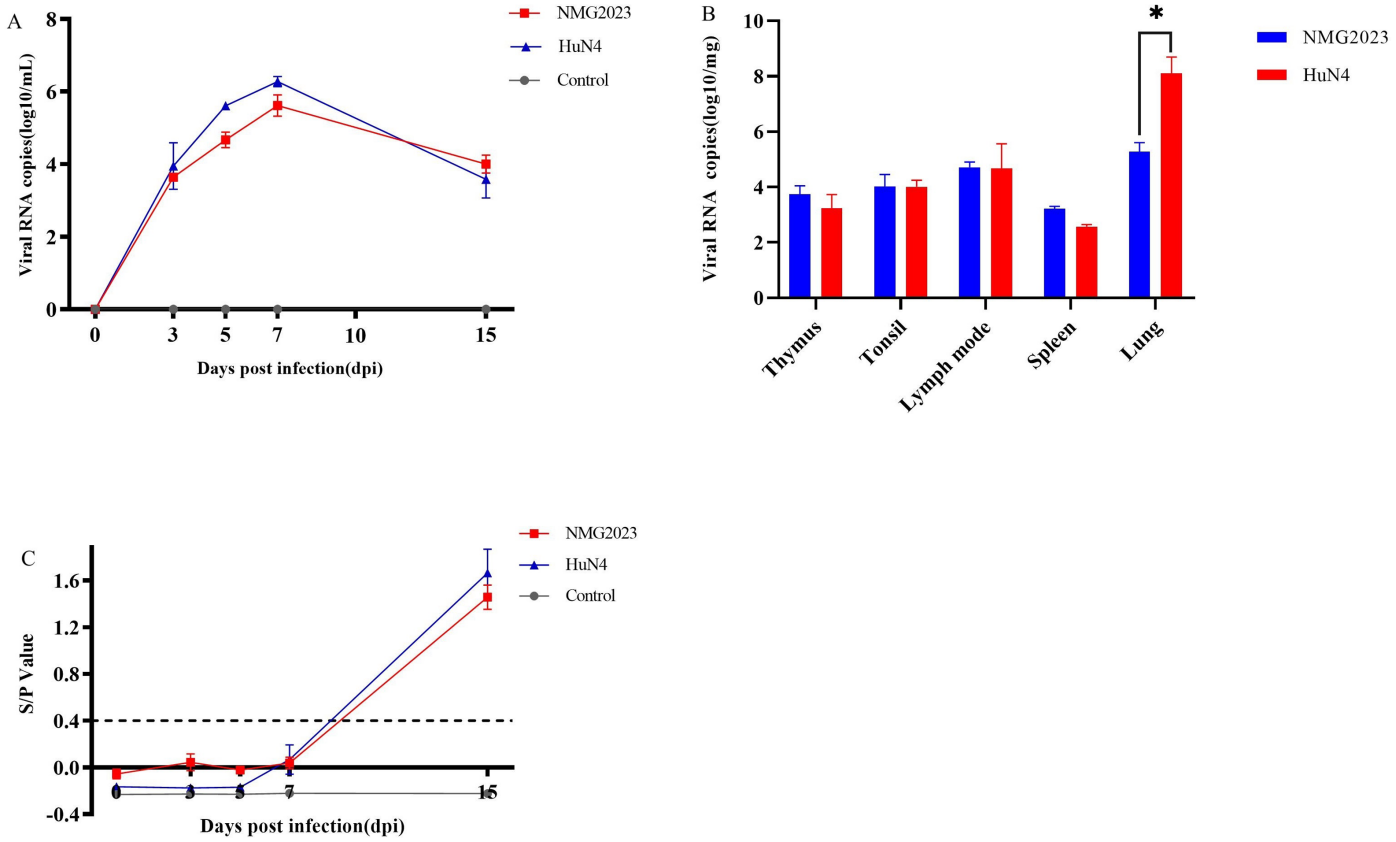
Detection of PRRSV viral load in serum, in tissue and antibody levels in serum, respectively. (A) PRRSV viral load in serum samples collected at 0, 3, 5, 7, 10, and 15 dpi was assessed via RT-qPCR. (B) PRRSV viral load in tissue samples collected at 15 dpi was determined by RT-qPCR. (C) PRRSV-specific antibodies in piglet serum, collected at 0, 3, 5, 7, 10, and 15 dpi, were measured using the PRRSV Ab Antibody Detection kit (JNT Company). Antibody levels are shown as the S/P ratio, with samples considered positive at S/P > 0.4. Significant differences of viral RNA copies in lung between SDVD-NMG2023-infected and HuN4-infected groups are indicated at *p < 0.05. Data are presented as mean ± standard deviation (error bars).

Using RT-qPCR, viremia was detected in both PRRSV-infected groups from 3 to 15 dpi, with no viremia observed in the control group (Figure 5A). While HuN4-infected piglets exhibited a higher viral load in serum than the SDVD-NMG2023 group, both infected groups displayed a similar viral load trend, peaking at 7 dpi and gradually declining toward the end of the study (Figure 5A). At 15 dpi, viral loads in tissues (thymus, lungs, lymph nodes, tonsils, and spleen) were also measured, with PRRSV detected in all examined tissues of both infected groups. Notably, the lung viral load in SDVD-NMG2023-infected piglets was lower than that in HuN4-infected piglets, though viral loads in other tissues showed no significant differences between the groups (Figure 5B).

PRRSV-induced antibody levels against the N protein were assessed at 0, 3, 5, 7, and 15 dpi using the PRRSV Antibody Detection Kit (JNT Company). Antibody responses began at 7 dpi in both infected groups, reaching an S/P value of 1.39 by 15 dpi, with no significant differences between the two PRRSV strains in their capacity to induce antibodies against the N protein (Figure 5C). No antibodies were detected in the negative control group.

## 4 Discussion

Recombination events between NADC30 and Chinese HP-PRRSV strains led to the emergence of several NADC30-like strains in China during 2015–2016, which exhibited more complex virulence profiles than the original NADC30 strain (9, 22–24). Current commercial vaccines have shown declining efficacy in providing sufficient cross-protection, particularly against NADC30-related PRRSV strains. For instance, the FJ1402 strain, reconstituted from NADC30 and HP-PRRSV, is highly pathogenic in piglets (22). Although commercial PRRSV vaccines, such as TJM-F92 and R98, offer partial protection against FJ1402, they do not provide complete immunity (22). Consequently, pathogenicity studies on recombinant PRRSV strains are essential to fully understand their characteristics, though live vaccines pose an added risk of promoting new recombinant strains in the field.

These recombinant strains have intensified the challenges of PRRS prevention and control in pig farms. The recombination of multiple epidemic PRRSV strains adds further complexity to effective PRRS management strategies. In this study, the PRRSV variant strain SDVD-NMG2023 was isolated from a PRRS-affected pig farm, where primarily 50- to 70-day-old pigs exhibited a 15% mortality rate. The whole-genome sequence analysis revealed that the isolate was recombined from three clinical epidemic strains (JXA1, NADC30, and NADC34), which are currently under presented using this recombinant configuration. Pathogenicity experiments indicated that the clinical signs induced by SDVD-NMG2023 were similar to field observations but milder, suggesting that the clinical manifestation of this PRRSV variant may depend on multiple interacting factors.

The SDVD-NMG2023 strain was identified as a recombinant based on the NADC30 PRRSV sequence, with the highest nucleotide identity of 89.8% with NADC30 PRRSV. It contains three non-continuous 131-aa deletion patterns in the NSP2 gene and shares the highest homology with NADC30 in the regions NSP2, NSP3, NSP9 to NSP12, ORF6, and ORF7. Conversely, the ORF5 gene exhibits 95.2% homology with NADC34 PRRSV, while regions NSP1 and NSP3 to NSP8 show the greatest homology to JXA1. These results indicate that multiple PRRSV strains may coexist within some pig herds, creating increased opportunities for viral recombination—a process essential for viral evolution, virulence modulation, and immune evasion (2). To further clarify the virulence characteristics of SDVD-NMG2023, an animal experiment was conducted to assess its pathogenicity in piglets, using the HP-PRRSV HuN4 strain as a comparative benchmark due to its well-documented clinical symptoms.

In piglet pathogenicity studies, Chinese NADC34-like PRRSV strains have demonstrated considerable variability in pathogenicity. For instance, the 1–7-4 PRRSV line, isolated in 2018, showed high genomic similarity to IA/2014/NADC34 (GenBank: MF326985.1) and was classified as an NADC34-like strain (10). Since then, NADC34-like strains have spread across major pig production regions in China, but clinical signs have varied among different strains. Following inoculation with the NADC34-like strain HLJDZD32-1901, isolated in Heilongjiang Province in 2019, pigs displayed mild respiratory symptoms and slight increases in body temperature, indicating HLJDZD32-1901 is a mild pathogenic strain in piglets (10, 13). In contrast, the JS2021NADC34 strain is highly pathogenic, causing persistent fever and mortality in piglets (11, 23).

In this study, piglets infected with SDVD-NMG2023—a recombinant strain derived from three clinical epidemic PRRSV strains—exhibited mild symptoms, with no significant changes in body temperature, mild lung lesions, and minimal impact on growth. These results were consistent with observations from HLJDZD32-1901 infections but differed from the more severe effects seen in piglets infected with JS2021NADC34 (10, 11, 13, 23).

This study isolated a novel PRRSV strain, SDVD-NMG2023, resulting from the recombination of three PRRSV strains (JXA1, NADC30, and NADC34). Phylogenetic and genomic characterizations were conducted, alongside an animal study to further assess its pathogenicity and potential field impact. SDVD-NMG2023-infected piglets exhibited serum viral loads and replication patterns comparable to those of HuN4, indicating rapid transmission capability within herds and across regions. Despite the absence of mortality among piglets during the pathogenicity study, thymic atrophy, lung pathological lesions, and weight loss were found in piglets inoculated with SDVD-NMG2023 strain. The novel strain may potentially impair the maturation of immune and respiratory system in infected piglets, resulting in considerable economic losses.

Since the identification of NADC30/34 strains on Chinese swine farms, recombination events involving both NADC30/34 and HP-PRRSV-like strains have continued to arise in swine populations (8, 11, 13, 24–27). Such multi-strain recombinations present considerable challenges to PRRS management, as current commercial vaccines often provide only partial or insufficient protection against these recombinants (28–30). Isolated from a PRRSV-positive swine farm undergoing vaccination, SDVD-NMG2023 was identified as a new recombinant virus of NADC30-like, NADC34-like, and JXA1-like strains. These findings highlight the urgent need for targeted control strategies and intervention measures to mitigate the spread and impact of such recombinant strains.

## 5 Conclusion

The novel PRRSV isolate SDVD-NMG2023, derived from 65-day-old pigs in Inner Mongolia, represents a natural recombination event among NADC30-like, NADC34-like, and JXA1-like strains, inducing atypical clinical manifestations compared to HP-PRRS and exerting immunosuppressive effects on swine herds.

## Data Availability

The datasets presented in this study can be found in online repositories. The name of the repository and accession number can be found at: www.ncbi.nlm.nih.gov/nuccore/, PQ644630.

## Footnotes

^1^ http://blast.ncbi.nlm.nih.gov
